# Why Don’t You Write?

**Published:** 1879-09-20

**Authors:** 


### EDITORIAL AND MISCELLANEOUS.

### *WHY DON'T YOU WRITE?*

We here repeat what we have often said to our subscribers, and the same is said to the members of the profession everywhere: we want medical facts and information for publication in THE RECORD. We have, it is true, elicited much practical information from the ranks of the profession. We wish it understood that while we have often received and shall yet expect articles from prominent and distinguished writers, our pages are also freely opened to the humblest member in the profession. Even those who are not blessed with high educational advantages, practical and sensible men, in the villages or at the cross roads, are requested to write us their views.

Among this class we not unfrequently find men who have been eminently successful as practitioners, oftentimes succeeding in emergencies which would appal those of higher attainments in the profession. In this we have evidence that there are facts and resources in the possession of such men not generally known. Let them come out.

We request, however, that all communications be brief, concise and practical.

